# Bacillus “next generation” diagnostics: moving from detection toward subtyping and risk-related strain profiling

**DOI:** 10.3389/fmicb.2013.00032

**Published:** 2013-02-22

**Authors:** Monika Ehling-Schulz, Ute Messelhäusser

**Affiliations:** ^1^Institute of Functional Microbiology, Department of Pathobiology, University of Veterinary Medicine ViennaVienna, Austria; ^2^Bavarian Health and Food Safety AuthorityOberschleißheim, Germany

**Keywords:** *Bacillus cereus*, *Bacillus* toxin, enterotoxins, cereulide, toxin gene profiling, MLST, fingerprinting, food poisoning

## Abstract

The highly heterogeneous genus *Bacillus* comprises the largest species group of endospore forming bacteria. Because of their ubiquitous nature, *Bacillus* spores can enter food production at several stages resulting in significant economic losses and posing a potential risk to consumers due the capacity of certain *Bacillus* strains for toxin production. In the past, food microbiological diagnostics was focused on the determination of species using conventional culture-based methods, which are still widely used. However, due to the extreme intra-species diversity found in the genus *Bacillus*, DNA-based identification and typing methods are gaining increasing importance in routine diagnostics. Several studies showed that certain characteristics are rather strain-dependent than species-specific. Therefore, the challenge for current and future *Bacillus* diagnostics is not only the efficient and accurate identification on species level but also the development of rapid methods to identify strains with specific characteristics (such as stress resistance or spoilage potential), trace contamination sources, and last but not least discriminate potential hazardous strains from non-toxic strains.

## INTRODUCTION

Proper diagnostic tools are of utmost importance, not only in the field of clinical but also in the field of food and veterinary microbiology diagnostics. Since the first isolation, purification, and cultivation of a pathogenic bacterium, namely *Bacillus anthracis*, by Koch (1876), the use of solid plating media has become the “golden standard” in classical microbiology and is still the “method of choice” for identification and enumeration of bacteria in routine diagnostic labs. However, general drawbacks of conventional culture-based methods, such as low specificity (poor inclusivity/exclusivity) and low discriminatory power, question their suitability to cope with today’s diagnostic needs. In addition, it has been show that certain characteristics, such as the presence of toxin genes, are rather strain than species-specific. For instance, botulinum neurotoxins are not only produced by *C. botulinum* but also by some *C. baratii* and *C. butyricum* strains, *Staphylococcus aureus* enterotoxin genes have also been found in other *Staphylococcus* spp. (e.g., Tsukamoto et al., 2002; De Medici et al., 2009; Oliveira et al., 2010). There are also some studies reporting on the detection of *B. cereus* enterotoxins in non-*B. cereus* group *Bacillus* spp. (Rowan et al., 2001; Phelps and McKillip, 2002) and, more recently, a heat stable toxin, structural related to the *B. cereus* emetic toxin cereulide, has been found in a *Paenibacillus tundrae* strain (Rasimus et al., 2012). The capacity for the production of spoilage-associated enzymes, such as proteases and lipases, may also vary significantly among strains of the same species (see, e.g., De Jonghe et al., 2010). Generally, members of the genus *Bacillus* and related genera show a high inter- and intra-species heterogeneity, which confronts diagnostic labs with various challenges and the urgent need for novel diagnostic concepts.

The most prominent member of the genus *Bacillus* is the *B. cereus* group that comprises several genetically closely related species, which are summarized under the term *Bacillus cereus sensu lato* (Kolsto et al., 2009; Ehling-Schulz et al., 2011a). With the increase of genetic information and the description of more and more so-called “borderline” strains (Klee et al., 2006; Fricker et al., 2008), a refinement of the current nomenclature might become necessary. Due to their different risk potentials, ranging from risk group 1 to risk group 3, the members of the *B. cereus* group are still handled as separate species. According to their major virulence characteristics, the following species are discriminated: *B. anthracis* that causes the fatal human and animal disease anthrax, *B. thuringiensis* that is commercially used as biopesticide (Mock and Fouet, 2001; Bravo et al., 2011), and *B. cereus sensu stricto*, the name giving species of the group. The latter one is an opportunistic human pathogen that can cause two forms of food poisoning: emesis and diarrhea. The diarrheal form of *B. cereus* food poisoning resembles the symptoms of a *C. perfringens* infection and the emetic form parallels the symptoms of intoxications related to *S. aureus* enterotoxins (for review, see Ehling-Schulz et al., 2004a; Stenfors Arnesen et al., 2008). Normally, both forms of the food borne disease are self-limited but reports on more severe cases, requiring hospitalization and including even death, are currently increasing (e.g., Lund et al., 2000; Dierick et al., 2005; Naranjo et al., 2011; Ehling-Schulz and Messelhaeusser, 2012). In addition, *B. cereus* can also cause non-gastrointestinal diseases, such as local eye or wound infection, as well as systemic infections, such as bacteraemia and endocarditis (for review, see Bottone, 2010). *B. mycoides* and *B. weihenstephanensis* are two psychrotolerant members of the group, which can grow even under refrigerator conditions. The latter two are known for their spoilage potential but their pathogenic potential seems to be rather low (Guinebretière et al., 2008, 2010). Very recently, *B. cytotoxicus*, a novel thermotolerant member of the group has been described, which may also possess food poisoning potential (Guinebretière et al., 2013).

Because of the medical, food safety and food quality relevance as well as economic importance, several methods for typing of *B. cereus s.l.* have been developed and comprehensive genome sequencing projects are underway, opening new avenues for diagnostics. This review will provide an overview on state of the art methods for identification and typing of this interesting group of spore formers and will discuss future trends in *Bacillus* diagnostics, focusing rather on strain characterization than on species identification.

## WHO IS OUT THERE?

**FIGURE 1:**
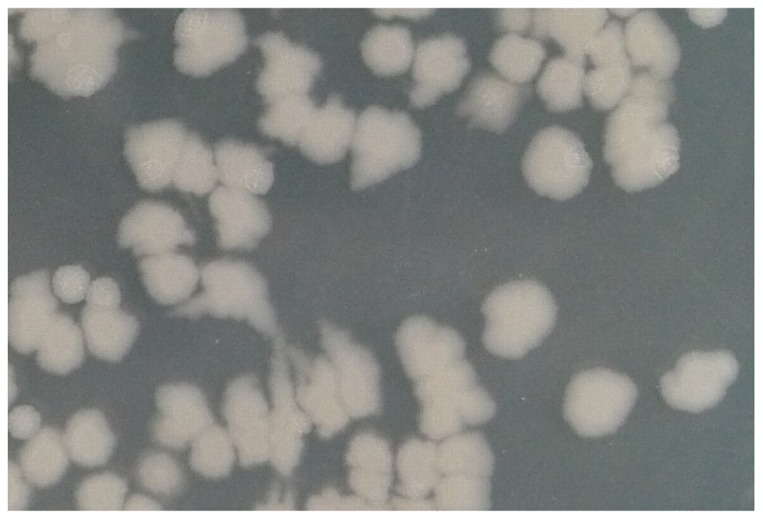
**Who is who? – a mixed culture of *B. cereus* group strains, namely: *B. thuringiensis* IEBC-T066001 (serotype entomocidus biovar subtoxicus, Canada), *B. weihenstephanensis* WSBC 10207 (isolated from pasteurized milk, Germany), and *B. cereus* F4430 (isolated from pea soup connected to a foodborne outbreak in Belgium)**.

The *B. cereus* group (**Figure 1**) comprises bacteria that can grow over a wide temperature range and showing quite variable pathogenic potentials, ranging from strains used as plant growth promoters and biopesticides to strains causing fatal diseases.

### THE CLASSICAL WAY OF *Bacillus* DIAGNOSTIC: CULTURAL DETECTION, ENUMERATION, AND DIFFERENTIATION

Cultural detection and enumeration of presumptive *B. cereus* (*B. cereus s.l*.), following internationals standards (such as the ISO 7932; Anonymous, 2004), is still the standard procedure for *Bacillus* diagnostic in food microbiology laboratories. Because of their close genetic relatedness, *B. cereus* group members cannot be differentiated by classical cultural detection methods or 16S rDNA sequencing and are therefore subsumed as “presumptive *B. cereus*.”

Isolation and enumeration of presumptive *B. cereus* from different food matrices is routinely performed using selective plating media. Two standard plating media, the polymyxin-egg yolk-mannitol-bromothymol blue agar (PEMBA) and the mannitol-egg yolk-polymyxin (MYP) agar, are currently recommended by the International Organization for Standardization (ISO) or the Food and Drug Administration (FDA). However, these media bear the risk of substantial misidentifications since various strains, especially if food matrices are analyzed, are showing atypical reaction on these media (Fricker et al., 2008). In the last few years, new chromogenic media have been designed for the detection of *B. cereus* group members. All these media are based on enzymes that are under regulatory control of the pleiotropic regulator PlcR. However, since molecular polymorphisms in the *plcR* gene have been found in all strains showing atypical growth characteristics, the concept of the selective plating media using PlcR-regulated enzymatic activities must be generally questioned and should be reconsidered (Fricker et al., 2008).

For the enumeration of presumptive *B. cereus* two main methods are widely used in food microbiology, colony-count-techniques on different solid agars and the most probable number (MPN)-technique. For colony count methods employing solid media the detection limit is routinely assessed according to international standards, such as the ISO 7932 (Anonymous, 2004). Since routinely 0.1 mL of the sample (fluid material) or the first serial dilution step (solid material) are plated, the theoretical detection limit is about 10 or 100 CFU/g, respectively, but the detection limit can be lowered by the power of ten by plating 1 mL of the sample or first dilution step on three plates of the solid medium. Enumeration procedures can also be combined with a molecular-based differentiation of the isolates based on their toxin gene profiles, enabling a rough estimate of the number of pathogenic *B. cereus* in the food sample investigated (see following section for details). Since *B. cereus* is a ubiquitous spore former, its presence cannot totally be avoided in many food products and, from a consumer safety perspective, the determination of the presence and prevalence of toxigenic strains is of special importance. First assays for enumeration of presumptive and/or toxigenic *B. cereus* strains employing molecular methods, such as qPCR have been described (Martínez-Blanch et al., 2009; Ceuppens et al., 2010; Dzieciol et al., 2013) but their applicability in route diagnostic is still hampered by the fact that the current systems do not allow a differentiation of live and dead bacteria, vegetative cells or spores.

### THE GOOD, THE BAD, THE UGLY: MOLECULAR TOOL BOX FOR TYPING AND PROFILING OF STRAINS

Molecular diagnostic tools for *B. cereus* focus more and more on detection of toxin genes rather than on the differentiation between *B. cereus sensu stricto* and other members of the *B. cereus* group. Although for other purposes the determination of the species might be required. Due to the bioterrorism potential *B. anthracis* various PCR systems for its specific detection and differentiation from the other members of the *B. cereus* group have been developed. For instance, Leski et al. (2009) used the *Bacillus* collagen-like protein *bcl* genes as target sequence whereas Wielinga et al. (2011) integrated a chromosomal marker sequence, target genes located on the two *B. anthracis* virulence plasmids and an internal amplification control in a probe-based multiplex real-time PCR assay. Since the latter assay targets, beside a *B. anthracis*-specific chromosomal marker, the coding region of the edema factor gene (*cya*; encoding an anthrax toxin component) located on pXO1, and the coding region of the capsule synthesis gene *capB*, located on pXO2, this assay allows a one-step detection and discrimination of different *B. anthracis* virulence types (Wielinga et al., 2011). Because of the economic importance of *B. thuringiensis* as biopesticide, PCR systems targeting different parts of the *cry* insecticidal toxin genes, have been developed during the last two decades (see, e.g., Bourque et al., 1993).

More recently, PCR systems for toxin gene profiling of *B. cereus* group strains have been developed. The detection of the different toxin genes can either be performed using gel-based PCR or real-time PCR systems (e.g., Guinebretière et al., 2002; Ehling-Schulz et al., 2004b, 2006b; Fricker et al., 2007; Wehrle et al., 2010). Because members of the *B. cereus* group frequently possess the ability to produce more than one toxin, suitable diagnostic tools for toxin gene profiling should cover the genes encoding the three main enterotoxins, namely the non-hemolytic enterotoxin (Nhe), the hemolysin BL (Hbl), and cytotoxin K (CytK) as well as the emetic toxin cereulide synthetase genes *ces*. Nhe and Hbl are related three component toxins whereas CytK is single component protein toxin, belonging to the group of β-barrel toxins (for review, see Stenfors Arnesen et al., 2008). The emetic toxin cereulide is a cyclic heat stable depsipeptide produced by the non-ribosomal cereulide peptide synthetase (Ehling-Schulz et al., 2005a, 2006a). Current studies indicate that literarily all *B. cereus* group strains carry the *nhe* genes and most of the strains are also able to produce Nhe, although the levels of toxin production very significantly from strains to strains (Moravek et al., 2006; Stenfors Arnesen et al., 2008). Between 44 and 60% of *B. cereus* strains are able to produce the Hbl toxin (Ehling-Schulz et al., 2005b; Moravek et al., 2006). The ability for CytK production was found in about 40–85% of *B. cereus* isolates investigated so far (Ngamwongsatit et al., 2008; Stenfors Arnesen et al., 2008). Generally, it seems that certain toxin gene profiles are predominant in specific groups of strains derived from different origin (Ehling-Schulz et al., 2005b, 2006b). For instance, *cytK* was found in 70% of strains connected to diarrheal food borne outbreaks but it was only rarely found in emetic strains (8%). Recent studies from different continents including isolates from diverse origins indicate the progressive emergence of pathotypes with novel toxin gene profiles (Thorsen et al., 2006; Ehling-Schulz et al., 2011a; Chon et al., 2012), confronting food industry and food microbiology labs with potential novel hazards.

For outbreak investigations of *B. cereus s.l.*, the toxin gene profile might be much more important than the exact species determination. It is well known that not only *B. cereus sensu stricto* can harbor the toxin genes described above; instead, enterotoxin genes are broadly distributed within the *B. cereus* group (e.g., Prüss et al., 1999; Guinebretière et al., 2008) and *B. thuringiensis* has also been described as the possible cause of foodborne outbreaks and other infections, such as local wound and eye infection as well as pulmonary infections (Jackson et al., 1995; Hernandez et al., 1998; Callegan et al., 2006; Ghelardi et al., 2007). Thorsen et al. (2006) reported on a *B. weihenstephanensis* strain carrying the cereulide synthetase genes and possessing the capability for cereulide toxin formation. Therefore, future developments in food microbiology diagnostics should be more focused on the determination of toxins and virulence factors than on the differentiation of species. Nevertheless, one should bear in mind that the sole presence or absence of an individual toxin gene does not fully explain the pathogenic potential of a certain strain and molecular methods should always be accompanied by sensitive and accurate toxin quantification systems (see, e.g., Bauer et al., 2010). For instance, it has been shown that the toxigenic potential among emetic as well as enterotoxic strains can vary substantially (see, e.g., Moravek et al., 2006; Stark et al., 2013).

## POPULATION STUDIES AND CONTAMINATION ROUTE ANALYSIS

### DIGGING INTO *Bacillus* POPULATIONS: PCR-BASED TYPING SYSTEMS

For molecular typing of members of the *B. cereus* group various PCR-based methods are currently available. Beside random amplification of polymorphic DNA (RAPD)-PCR, REP (repetitive extragenic palindromic)-, ERIC (enterobacterial repetitive intergenic consensus)-, or BOX-PCR can be used for genomic fingerprinting of isolates. For instance, BOX-PCR genomic fingerprinting and also variable-number tandem repeats (VNTR) analysis show the close relationship between *B. anthracis* and some *B. cereus* strains (e.g., Kim et al., 2002; Chaves et al., 2011). Since several years, the RAPD-PCR is an established method for molecular typing of different members of *Bacillus* spp. For instance, RAPD was applied for epidemiological subtyping of *B. cereus* and *B. lentus* and for differentiation between *B. anthracis* and other members of the *B. cereus* group (Stephan, 1996; Daffonchio et al., 1999). RAPD may also represent an interesting screening method for emetic *B. cereus* strains also on a routine laboratory basis (Ehling-Schulz et al., 2005b). In summary, RAPD is a valuable and widely used tool for molecular typing of different *Bacillus* spp. Nevertheless, in contrast to other molecular typing methods, such as multilocus sequence typing (MLST), the interlaboratory reproducibility of data frequently causes difficulties, which might have been one of the reasons why MLST-based systems gradual became the “golden standard” during the last years.

### THE “GOLDEN STANDARDS” FOR POPULATION STUDIES OF *B. Cereus s.l.*: MULTILOCUS SEQUENCE TYPING AND AMPLIFIED FRAGMENT LENGTH POLYMORPHISM

Due to high genomic plasticity, the population structure of *B. cereus s.l.* is quite dynamic and there is potential within this group of bacteria for emergence of new pathogenic lineages with increased or new virulence, or increased ability to survive in adverse environmental conditions (Kolsto et al., 2009; Ehling-Schulz et al., 2011a; Tourasse et al., 2011). An in-depth knowledge of the population structure of *B. cereus s.l.* is therefore not only of general academic interest but also of great importance for clinical and food microbiology diagnostics. During the last decade various MLST-based schemes for typing of *B. cereus s.l.* strains have been developed (for overview, see Tourasse and Kolsto, 2008), which have been successfully applied for inferring genetic relationships among *B. cereus s.l.* strains of different origin, such as soil, insects, food, and humans (Ehling-Schulz et al., 2005b; Vassileva et al., 2006; Cardazzo et al., 2008; Hoffmaster et al., 2008; Raymond et al., 2010). Although the different MLST schemes employ different house keeping genes, all of them revealed three major clades. Interestingly, the same major clusters were found by Fourier transform infrared (FTIR) spectroscopic analysis, pointing toward conserved phenotypic traits of genetic-related strains (Ehling-Schulz et al., 2005b). However, regardless of the typing method used, the different *B. cereus* group species are interspersed within the different clusters, questioning the suitability of diagnostics solely based on species identification.

One major drawback of MLST-based approaches is the requirement of substantial hands-on-time for sequencing of seven genes per strain and subsequent data analysis, which limits its applicability for high throughput studies. The development of microfluidic biochips might simplify MLST analysis in the future (Read et al., 2010). Currently, the use of the sporulation stage III AB gene (*spoIIIAB*) as a single genetic marker might represent an alternative to obtain a rough snapshot of genetic relations among *B. cereus s.l.* strains under study (Ehling-Schulz et al., 2005b). This genetic marker resembles the structure of MLST-derived clusters and its suitability for sequence typing was recently reconfirmed by comparing clusters derived form hierarchical cluster analysis of *spoIIIAB* sequences with the clusters obtained by whole genome sequencing using a sliding window approach (Fricker et al., 2011; Segerman et al., 2011).

When high throughput capacities are needed, amplified fragment length polymorphism (AFLP) might be the method of choice because it does not require laborious sequencing efforts. For instance, Guinebretière et al. (2008) used AFLP for typing of a comprehensive collection of 425 well-characterized *B. cereus* group strains derived from very different ecological niches. Seven major clusters (denoted I–VII) were identified, which correlate with physiological properties of the strains. Interestingly, the potential of strains for causing food poisoning correlated with certain phylogenetic groups (Guinebretière et al., 2010). To assign strains to different genetic groups an online tool has been developed, which is available at https://www.tools.symprevius.org/Bcereus/english.php.

In addition, Tourasse et al. (2010) have developed a database called HyperCat, allowing the integration of data from the two different typing systems (MLST, AFLP) described above as well as data derived from multilocus enzyme electrophoresis (MEE), to calculate super trees. HyperCat was applied to carry out a multi-data type analysis on 2213 strains of different origin, including 450 food and dairy production strains. This integrative approach confirmed the major clusters but also revealed some novel phylogenetic branches, including a putative new lineage of *B. anthracis* (Tourasse et al., 2011). The next step toward a more holistic understanding of this evolutionary interesting and economical important group of microorganisms would be now to include data from functional genomics (transcriptomics, proteomics, and metabolomics).

### BACTERIAL IDs: FINGERPRINTING TECHNIQUES

#### Molecular fingerprints

The main fingerprinting technique, the pulsed field gel electrophoresis (PFGE) is used as one of the most important typing method for a wide field of foodborne pathogens, especially for epidemiological studies in outbreak situations. In principle, PFGE can be used for typing of *B. cereus* (Carlson et al., 1994; Liu et al., 1997; Ohsaki et al., 2007) but PulseNet International, a network for tracking foodborne infections worldwide, does not provide a protocol for molecular typing of *B. cereus* so far (Swaminathan et al., 2006). However, for epidemiological studies, especially in case of foodborne outbreaks, standard protocols would be mandatory for generating comparable data worldwide. In addition, there are technical difficulties in attaining sufficient chromosomal DNA for macrorestriction of certain strains, especially from food-derived ones. Generally, MLST and AFLP are more commonly used for the differentiation and epidemiological investigations of *B. cereus* group members than PFGE, and multilocus VNTR analysis (MLVA) and single-nucleotide polymorphism (SNP) analysis are the current “methods of choice” for typing of isolates belonging to the highly monomorphic species *B. anthracis* (e.g., Keim et al., 2000; Kuroda et al., 2010).

#### Metabolic fingerprints

Fourier transform infrared spectroscopy is a powerful tool for microbial diagnostics and epidemiological studies and has already been successfully used to type *B. cereus* group strains (Ehling-Schulz et al., 2005b; Mietke et al., 2010). Basically, FTIR is a vibrational spectroscopic technique, which is able to distinguish microbial cells at different taxonomic levels (Naumann et al., 1991; Wenning et al., 2008). The entire biochemical composition of whole cells is recorded by the absorbance of mid-infrared light by the molecules present in the cells. The resulting spectra are used as fingerprints and analyzed by pattern recognition techniques. The same spectrum from a microbial sample can be used for identification purposes as well as for typing below the species level. This enables an application in contamination route analysis, epidemiological studies and for determination of specific properties of *B. cereus s.l.* (Ehling-Schulz et al., 2005b, 2011b). Due to its cost efficiency and high throughput capacities, FTIR spectroscopy represents an interesting alternative to genetic methods for *B. cereus* subtyping and for tracing contamination sources.

## THIRD GENERATION SEQUENCING FOR NEXT GENERATION DIAGNOSTICS?

The introduction of massive parallel sequencing in the mid-2000s was a hallmark in genome sequencing, allowing rapid sequencing of DNA on a gigabase scale. The advances in high throughput sequencing technologies during the last years enable the sequencing of microbial genomes in less than 1 day. Concurringly with the upscaling of sequencing capacities the costs per base for sequencing are constantly dropping, thereby opening new perspectives for genome-based diagnostics.

The role of *B. anthracis* as a potent bioterror agent has lead to a renewed interest in its close relative *B. cereus*, resulting in several genome sequencing projects. Currently (January 2013), genomic sequence information is available for about 225 *B. cereus* group strains (**Table 1**). The list of strains in the sequencing pipeline is steadily growing but the lack of bioinformatic tools is still the bottleneck for a broader application of genome sequence-based diagnostics. Especially for bacteria, such as *B. cereus*, showing a high rate of genome rearrangements and genomic repeats and transposable elements (Tourasse et al., 2006; Kolsto et al., 2009; Ehling-Schulz et al., 2011a) *de novo* sequence assembly is laborious and time consuming. Therefore, more and more genomes are left unfinished as permanent draft sequences. Third generation sequencing may help to overcome this obstacle to a certain degree by generating longer reads, facilitating sequence assembly. However, the development of appropriate, user-friendly bioinformatic tools will be the major challenge for implementation of genomics in microbial diagnostics in the upcoming years.

**Table 1:** Overview on *Bacillus* species for which multiple genomes are public available (http://www.ncbi.nlm.nih.gov/genome, last accessed January 2013)

Species	Number of genomes Σ*	Economic relevance
***B. cereus sensu lato***
*Bacillus cereus*	154 (41)	P, S, (B)
*Bacillus thuringiensis*	32 (23)	B, (P)
*Bacillus anthracis*	31 (7)	P
*Bacillus weihenstephanensis*	5 (1)	S
*Bacillus mycoides*	3 (3)	S
**Non-*B. cereus*** **group *Bacillus* spp.**
*Bacillus subtilis*	42 (13)	B, S, (P)
*Bacillus amyloliquefaciens*	19 (10)	B
*Bacillus atrophaeus*	16 (1)	§
*Bacillus licheniformis*	10 (2)	B, (S), (P)
*Bacillus megaterium*	7 (4)	B
*Bacillus pumilus*	7 (1)	B, (S), (P)
*Bacillus coagulans*	5 (2)	B, S

* Number refers to *Bacillus* genome sequencing projects; number of finished genomes is given in brackets.

P, pathogenic potential; S, spoilage potential; B, beneficial (biopesticide, probiotic, plant growth promoter; biotechnological applications).

§ Use as non-pathogenic surrogate in food microbiology and biodefense research.

First tools to minimize post-sequencing data processing have already been developed. For instance, Segerman et al. (2011) used a method that defines orthologous sequence reads instead of orthologous genes for subtyping *B. anthracis* strains and for obtaining a general overview of the phylogenomic structure of the genus *Bacillus*. Very recently, Agren et al. (2012) presented a software tool, which uses fragmented alignments to analyze multiple genomes. This software, named after the Greek Argonauts six-armed giant tribe “Gegenees,” is designed as an open platform and can be accessed at http://gegenees.org/index.html. “Gegenees” was successfully used to search for unique signatures for *B. anthracis*, by analyzing 134 *Bacillus* genomes. Based on the identified signatures, target group specific primers were designed (Agren et al., 2012).

For comparative genomotyping DNA microarray-based analysis might also be an interesting route to follow. The huge amount of genetic information from recent and ongoing *Bacillus* sequencing projects makes it feasible to design high-density whole genome microarrays to gain in-depth insights into genetic footprints of strains. For instance, Papazisi et al. (2011) used a multi-genome DNA array to study the genomic diversity of *B. cereus s.l.* and evolutionary traits of *B. anthracis*. Such arrays are not only useful to gain insights into the pathophysiology of Bacilli but might also be valuable tools to search for specific strain characteristics, such as stress resistance genes and spoilage-associated genetic determinants.

## CONCLUSION AND FUTURE PERSPECTIVES

The pathogenic potential among *B. cereus* group strains ranges from probiotics to highly toxic strains, causing fatal diseases. The discrimination of hazardous strains from harmless, or even beneficial, isolates is therefore the major challenge in future *B. cereus* diagnostics. It is expected that the currently taxonomic focused diagnostics will gradually be replaced/or complemented by more risk orientated diagnostics. The diagnostic tools, developed during the last decade, for toxin gene profiling and for the determination of specific molecular characteristics as well as for the detection of specific patho- and ecotypes and for quantification of toxins are gaining increasing importance and will lead to a significant improvement of *B. cereus* diagnostics.

**FIGURE 2:**
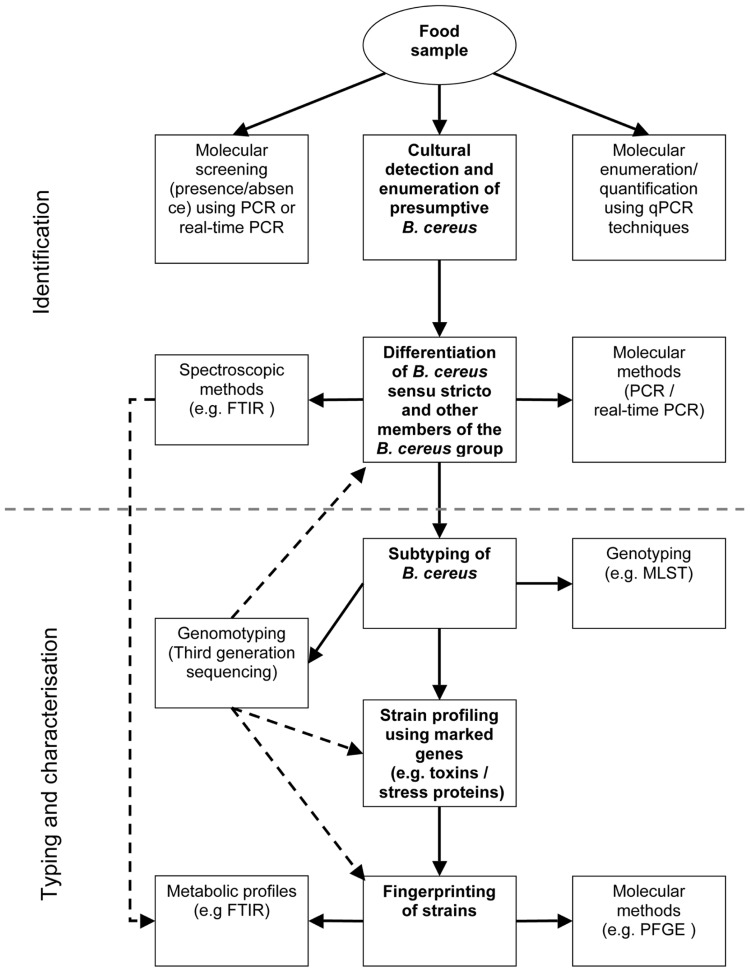
**Work flow for differential diagnostic of *B. cereus s.l.* in food samples.** - - - - : spectral/sequence data generated once can be used for multiple analyses.

However, the classical cultural methods for detection and enumeration of members of the *B. cereus* group are still important tools in the field of food microbiology and could complement and cross-validate results from molecular analyses (**Figure 2**). Because current methods exploited for identification and subtyping of *B. cereus s.l.* require the isolation of single strains, culture-based methods will still be an intrinsic part of food microbiology diagnostics during the next years.

Recent developments in genomotyping are also opening new perspectives for food microbiology diagnostics and are expected to (i) help to decipher specific molecular characteristics of highly pathogenic, food spoiling or beneficial strains and provide biomarkers for a new generation of diagnostics, (ii) foster rapid contamination route analyses, which is getting increasingly important due to globalization of food production, and (iii) facilitate tracing of sources of food borne outbreak by supporting linkage of patient isolates with food-derived isolates. However, before the full potential of next generation sequence-based genomics, or even a part of it, can be exploited for food microbiology diagnostic, appropriate user-friendly bioinformatic tools need to be developed.

## Conflict of Interest Statement

The authors declare that the research was conducted in the absence of any commercial or financial relationships that could be construed as a potential conflict of interest.

## References

[B1] Agren J. Sundström A. Håfström T. Segerman B. (2012). Gegenees: fragmented alignment of multiple genomes for determining phylogenomic distances and genetic signatures unique for specified target groups. *PLoS ONE* 7:e39107. 10.1371/journal.pone.0039107 PMC3377601 22723939

[B2] Anonymous. (2004). *Microbiology of Food and Animal Feeding Stuffs* – *Horizontal Method for the Enumeration of Presumptive Bacillus cereus* – *Colony-count Technique at 30 Degrees C*. Geneva: International Organization for Standardization (ISO)

[B3] Bauer T. Stark T. Hofmann T. Ehling-Schulz M. (2010). Development of a stable isotope dilution analysis for the quantification of the *Bacillus cereus* toxin cereulide in foods. *J. Agric. Food Chem.* 58 1420–1428 19994891 10.1021/jf9033046

[B4] Bottone E. J. (2010). *Bacillus cereus*, a volatile human pathogen. *Clin. Microbiol. Rev.* 23 382–438 20375358 10.1128/CMR.00073-09 PMC2863360

[B5] Bourque S. N. Valéro J. R. Mercier J. Lavoie M. C. Levesque R. C. (1993). Multiplex polymerase chain reaction for detection and differentiation of the microbial insecticide* Bacillus thuringiensis*. *Appl. Environ. Microbiol.* 59 523–527 8434916 10.1128/aem.59.2.523-527.1993 PMC202137

[B6] Bravo A. Likitvivatanavong S. Gill S. S Soberón M. (2011). *Bacillus thuringiensis*: a story of a successful bioinsecticide. *Insect Biochem. Mol. Biol.* 41 423–431 21376122 10.1016/j.ibmb.2011.02.006 PMC3689885

[B7] Callegan M. C. Cochran D. C. Kane S. T. Ramadan R. T. Chodosh J. McLean C. (2006). Virulence factor profiles and antimicrobial susceptibilities of ocular *Bacillus* isolates. *Curr. Eye Res.* 31 693–702 16966141 10.1080/02713680600850963

[B8] Cardazzo B. Negrisolo E. Carraro L. Alberghini L. Patarnello T. Giaccone V. (2008). Multiple-locus sequence typing and analysis of toxin genes in *Bacillus cereus* food-borne isolates. *Appl. Environ. Microbiol.* 74 850–860 18083872 10.1128/AEM.01495-07 PMC2227710

[B9] Carlson C. R. Caugant D. A Kolstø A. B. (1994). Genotypic diversity among *Bacillus cereus* and *Bacillus thuringiensis* strains. *Appl. Environ. Microbiol.* 60 1719–1725 16349267 10.1128/aem.60.6.1719-1725.1994 PMC201553

[B10] Ceuppens S. Boon N. Rajkovic A. Heyndrickx M. Van de Wiele T. Uyttendaele M. (2010). Quantification methods for *Bacillus cereus* vegetative cells and spores in the gastrointestinal environment. *J. Microbiol. Methods* 83 202–210 20849884 10.1016/j.mimet.2010.09.009

[B11] Chaves J. Q. Pires E. S. Vivoni A. M. (2011). Genetic diversity, antimicrobial resistance and toxigenic profiles of *Bacillus cereus* isolated from food in Brazil over three decades. *Int. J. Food Microbiol.* 147 12–16 21440319 10.1016/j.ijfoodmicro.2011.02.029

[B12] Chon J. W. Kim J. H. Lee S. J. Hyeon J. Y. Seo K. H. (2012). Toxin profile, antibiotic resistance, and phenotypic and molecular characterization of *Bacillus cereus* in Sunsik. *Food Microbiol.* 32 217–222 22850397 10.1016/j.fm.2012.06.003

[B13] Daffonchio D. Borin S. Frova G. Gallo R. Mori E. Fani R. (1999). A randomly amplified polymorphic DNA marker specific for the *Bacillus cereus* group is diagnostic for *Bacillus anthracis*. *Appl. Environ. Microbiol.* 65 1298–1303 10049896 10.1128/aem.65.3.1298-1303.1999 PMC91177

[B14] De Jonghe V. Coorevits A. De Block J. Van Coillie E. Grijspeerdt K. Herman L. (2010). Toxinogenic and spoilage potential of aerobic spore-formers isolated from raw milk. *Int. J. Food Microbiol.* 136 318–325 19944473 10.1016/j.ijfoodmicro.2009.11.007

[B15] De Medici D. Anniballi F. Wyatt G. M. Lindström M. Messelhäusser U. Aldus C. F. (2009). Multiplex PCR for detection of botulinum neurotoxin-producing clostridia in clinical, food, and environmental samples. *Appl. Environ. Microbiol.* 75 6457–6461 19684163 10.1128/AEM.00805-09 PMC2765140

[B16] Dierick K. Van Coillie E. Swiecicka I. Meyfroidt G. Devlieger H. Meulemans A. (2005). Fatal family outbreak of *Bacillus cereus*-associated food poisoning. *J. Clin. Microbiol.* 43 4277–4279 16082000 10.1128/JCM.43.8.4277-4279.2005 PMC1233987

[B17] Dzieciol M. Fricker M. Wagner M. Hein I. Ehling-Schulz M. (2013). A diagnostic real-time PCR assay for quantification and differentiation of emetic and non-emetic *Bacillus cereus* in milk. *Food Control* 32 176–185

[B18] Ehling-Schulz M. Fricker M. Grallert H. Rieck P. Wagner M. Scherer S. (2006a). Cereulide synthetase gene cluster from emetic *Bacillus cereus*: structure and location on a mega virulence plasmid related to *Bacillus anthracis* toxin plasmid pXO1. *BMC Microbiol.* 6:20. 10.1186/1471-2180-6-20 PMC1459170 16512902

[B19] Ehling-Schulz M. Guinebretiere M. H. Monthan A. Berge O. Fricker M. Svensson B. (2006b). Toxin gene profiling of enterotoxic and emetic *Bacillus cereus*. *FEMS Microbiol. Lett.* 260 232–240 16842349 10.1111/j.1574-6968.2006.00320.x

[B20] Ehling-Schulz M. Fricker M. Scherer S. (2004a). *Bacillus cereus*, the causative agent of an emetic type of food borne illness. *Mol. Nutr. Food Res.* 48 479–487 15538709 10.1002/mnfr.200400055

[B21] Ehling-Schulz M. Fricker M. Scherer S. (2004b). Identification of emetic toxin producing *Bacillus cereus* strains by a novel molecular assay. *FEMS Microbiol. Lett.* 232 189–195 15033238 10.1016/S0378-1097(04)00066-7

[B22] Ehling-Schulz M. Knutsson R. Scherer S. (2011a). “*Bacillus cereus*” in *Genomes of Food- and Water-Borne Pathogens,* edsKathariou S. Fratamico P. Liu Y. (Washington, DC: ASM Press) 147–164

[B23] Ehling-Schulz M. Messelhäusser U. Granum P. E. (2011b). “*Bacillus cereus* in milk and dairy production,” in *Rapid Detection, Characterization and Enumeration of Food-borne Pathogens,* ed.Hoorfar J. (Washington, DC: ASM Press) 275–289

[B24] Ehling-Schulz M. Messelhaeusser U. (2012). “One pathogen but two different types of food borne outbreaks; *Bacillus cereus* in catering facilities in Germany,” in *Case Studies in Food Safety and Quality Management: Lessons from Real-life Situations,* ed.Hoorfar J. (Cambridge: Woodhead Publishing) 63–70

[B25] Ehling-Schulz M. Vukov N. Schulz A. Shaheen R. Andersson M. Märtlbauer E. (2005a). Identification and partial characterization of the nonribosomal peptide synthetase gene responsible for cereulide production in emetic *Bacillus cereus*. *Appl. Environ. Microbiol.* 71 105–113 15640177 10.1128/AEM.71.1.105-113.2005 PMC544239

[B26] Ehling-Schulz M. Svensson B. Guinebretiere M.-H. Lindbäck T. Andersson M. Schulz A. (2005b). Emetic toxin formation of *Bacillus cereus* is restricted to a single evolutionary lineage of closely related strains. *Microbiology* 151 183–197 15632437 10.1099/mic.0.27607-0

[B27] Fricker M. Ågren J. Segerman B. Knutsson R. Ehling-Schulz M. (2011). Evaluation of *Bacillus* strains as model systems for the work on *Bacillus anthracis* spores. *Int. J. Food Microbiol.* 145 S129–S136 20800917 10.1016/j.ijfoodmicro.2010.07.036

[B28] Fricker M. Messelhäußer U. Busch U. Scherer S. Ehling-Schulz M. (2007). Diagnostic real-time PCR assays for the detection of emetic *Bacillus cereus* strains in foods and recent foodborne outbreaks. *Appl. Environ. Microbiol.* 73 1892–1898 17259359 10.1128/AEM.02219-06 PMC1828801

[B29] Fricker M. Reissbrodt R. Ehling-Schulz M. (2008). Evaluation of standard and new chromogenic selective plating media for isolation and identification of *Bacillus cereus.* *Int. J. Food Microbiol.* 121 27–34 18055052 10.1016/j.ijfoodmicro.2007.10.012

[B30] Ghelardi E. Celandroni F. Salvetti S. Fiscarelli E. Senesi S. (2007). *Bacillus thuringiensis* pulmonary infection: critical role for bacterial membrane-damaging toxins and host neutrophils. *Microbes Infect.* 9 591–598 17387030 10.1016/j.micinf.2007.02.001

[B31] Guinebretière M. H. Auger S. Galleron N. Contzen M. De Sarrau B. De Buyser M. L. (2013). *Bacillus cytotoxicus* sp.nov. is a new thermotolerant species of the *Bacillus cereus* group occasionally associated with food poisoning. *Int. J. Syst. Evol. Microbiol.* 6 31–40 22328607 10.1099/ijs.0.030627-0

[B32] Guinebretière M. H. Broussolle V. Nguyen-The C. (2002). Enterotoxigenic profiles of food-poisoning and food-borne *Bacillus cereus* strains. *J. Clin. Microbiol.* 40 3053–3056 12149378 10.1128/JCM.40.8.3053-3056.2002 PMC120679

[B33] Guinebretière M. H. Thompson F. L. Sorokin A. Normand P. Dawyndt P. Ehling-Schulz M. (2008). Ecological diversification in the *Bacillus cereus* group. *Environ. Microbiol.* 10 851–865 18036180 10.1111/j.1462-2920.2007.01495.x

[B34] Guinebretière M. H. Velge P. Couvert O. Carlin F. Debuyser M. L. Nguyen-The C. (2010). Ability of *Bacillus cereus* group strains to cause food poisoning varies according to phylogenetic affiliation (groups I to VII) rather than species affiliation. *J. Clin. Microbiol.* 48 3388–3391 20660215 10.1128/JCM.00921-10 PMC2937725

[B35] Hernandez E. Ramisse F. Ducoureau J. P. Cruel T. Cavallo J. D. (1998). *Bacillus thuringiensis subsp. konkukian* (serotype H34) superinfection: case report and experimental evidence of pathogenicity in immunosuppressed mice. *J. Clin. Microbiol.* 36 2138–2139 9650985 10.1128/jcm.36.7.2138-2139.1998 PMC105009

[B36] Hoffmaster A. R. Novak R. T. Marston C. K. Gee J. E. Helsel L. Pruckler J. M. (2008). Genetic diversity of clinical isolates of *Bacillus cereus* using multilocus sequence typing. *BMC Microbiol. * 8:191. 10.1186/1471-2180-8-191 PMC2585095 18990211

[B37] Jackson S. G. Goodbrand R. B. Ahmed R. Kasatiya S. (1995). *Bacillus cereus* and *Bacillus thuringiensis* isolated in a gastroenteritis outbreak investigation. *Lett. Appl. Microbiol.* 21 103–105 7639990 10.1111/j.1472-765x.1995.tb01017.x

[B38] Keim P. Price L. B. Klevytska A. M. Smith K. L. Schupp J. M. Okinaka R. (2000). Multiple-locus variable-number tandem repeat analysis reveals genetic relationships within *Bacillus anthracis*. *J. Bacteriol.* 182 2928–2936 10781564 10.1128/jb.182.10.2928-2936.2000 PMC102004

[B39] Kim W. Hong Y. P. Yoo J. H. Lee W. B. Choi C. S. Chung S. I. (2002). Genetic relationships of *Bacillus anthracis* and closely related species based on variable-number tandem repeat analysis and BOX-PCR genomic fingerprinting. *FEMS Microbiol. Lett.* 207 21–27 11886745 10.1111/j.1574-6968.2002.tb11022.x

[B40] Klee S. R. Ozel M. Appel B. Boesch C. Ellerbrok H. Jacob D. (2006). Characterization of *Bacillus anthracis*-like bacteria isolated from wild great apes from Cote d’Ivoire and Cameroon. *J. Bacteriol.* 188 5333–5344 16855222 10.1128/JB.00303-06 PMC1540047

[B41] Koch R. (1876). Untersuchungen über Bakterien: V. Die Ätiologie der Milzbrand-Krankheit, begründet auf die Entwicklungsgeschichte des *Bacillus anthracis.* *Cohns Beitr. Biol. Pflanz.* 2 277–310

[B42] Kolsto A. B. Tourasse N. J. Okstad O. A. (2009). What sets *Bacillus anthracis* apart from other *Bacillus* species? *Annu. Rev. Microbiol.* 63 451–476 19514852 10.1146/annurev.micro.091208.073255

[B43] Kuroda M. Serizawa M. Okutani A. Sekizuka T. Banno S. Inoue S. (2010). Genome-wide single nucleotide polymorphism typing method for identification of *Bacillus anthracis* species and strains among *B. cereus* group species. *J. Clin. Microbiol.* 48 2821–2829 20554827 10.1128/JCM.00137-10 PMC2916593

[B44] Leski T. A. Caswell C. C. Pawlowski M. Klinke D. J. Bujnicki J. M. Hart S. J. (2009). Identification and classification of *bcl* genes and proteins of *Bacillus cereus* group organisms and their application in *Bacillus anthracis* detection and fingerprinting. *Appl. Environ. Microbiol.* 75 7163–7172 19767469 10.1128/AEM.01069-09 PMC2786505

[B45] Liu P. Y. Ke S. C. Chen S. L. (1997). Use of pulsed-field gel electrophoresis to investigate a pseudo-outbreak of *Bacillus cereus* in a pediatric unit. *J. Clin. Microbiol.* 35 1533–1535 9163476 10.1128/jcm.35.6.1533-1535.1997 PMC229781

[B46] Lund T. De Buyser M. L. Granum P. E. (2000). A new cytotoxin from *Bacillus cereus* that may cause necrotic enteritis. *Mol. Microbiol.* 8 254–261 11069652 10.1046/j.1365-2958.2000.02147.x

[B47] Martínez-Blanch J. F. Sánchez G. Garay E. Aznar R. (2009). Development of a real-time PCR assay for detection and quantification of enterotoxigenic members of *Bacillus cereus *group in food samples. *Int. J. Food Microbiol.* 35 15–21 19665814 10.1016/j.ijfoodmicro.2009.07.013

[B48] Mietke H. Beer W. Schleif J. Schabert G. Reissbrodt R. (2010). Differentiation between probiotic and wild-type *Bacillus cereus* isolates by antibiotic susceptibility test and Fourier transform infrared spectroscopy (FT-IR). *Int. J. Food. Microbiol.* 140 57–60 20303194 10.1016/j.ijfoodmicro.2010.02.009

[B49] Mock M. Fouet A. (2001). Anthrax. *Annu. Rev. Microbiol.* 55 647–671 11544370 10.1146/annurev.micro.55.1.647

[B50] Moravek M. Dietrich R. Buerk C. Broussolle V. Guinebretière M. H. Granum P. E. (2006). Determination of the toxic potential of *Bacillus cereus* isolates by quantitative enterotoxin analyses. *FEMS Microbiol. Lett.* 257 293–298 16553866 10.1111/j.1574-6968.2006.00185.x

[B51] Naranjo M. Denayer S. Botteldoorn N. Delbrassinne L. Veys J. Waegenaere J. (2011). Sudden death of a young adult associated with *Bacillus cereus* food poisoning. *J. Clin. Microbiol.* 249 4379–4381 22012017 10.1128/JCM.05129-11 PMC3232990

[B52] Naumann D. Helm D. Labischinski H. (1991). Microbiological characterizations by FT-IR spectroscopy. *Nature* 351 81–82 1902911 10.1038/351081a0

[B53] Ngamwongsatit P. Buasri W. Pianariyanon P. Pulsrikarn C. Ohba M. Assavanig A. (2008). Broad distribution of enterotoxin genes (hblCDA, nheABC, cytK, and entFM) among *Bacillus thuringiensis* and *Bacillus cereus* as shown by novel primers. *Int. J. Food Microbiol.* 121 352–356 18068844 10.1016/j.ijfoodmicro.2007.11.013

[B54] Ohsaki Y. Koyano S. Tachibana M. Shibukawa K. Kuroki M. Yoshida I. (2007). Undetected *Bacillus* pseudo-outbreak after renovation work in a teaching hospital. *J. Infect.* 54 617–622 17145080 10.1016/j.jinf.2006.10.049

[B55] Oliveira A. M. Miya N. T. Sant’Ana A. S. Pereira J. L. (2010). Behavior and enterotoxin production by coagulase negative *Staphylococcus* in cooked ham, reconstituted skimmed milk, and confectionery cream. *J. Food Sci.* 75 M475–M481 21535559 10.1111/j.1750-3841.2010.01754.x

[B56] Papazisi L. Rasko D. A. Ratnayake S. Bock G. R. Remortel B. G. Appalla L. (2011). Investigating the genome diversity of *B. cereus* and evolutionary aspects of *B. anthracis* emergence. *Genomics* 298 26–39 21447378 10.1016/j.ygeno.2011.03.008 PMC3129444

[B57] Phelps R. J. McKillip J. L. (2002). Enterotoxin production in natural isolates of *Bacillaceae* outside the *Bacillus cereus* group. *Appl. Environ. Microbiol.* 68 3147–3151 12039781 10.1128/AEM.68.6.3147-3151.2002 PMC123918

[B58] Prüss B. M. Francis K. P. von Stetten F. Scherer S. (1999). Correlation of 16S ribosomal DNA signature sequences with temperature-dependent growth rates of mesophilic and psychrotolerant strains of the *Bacillus cereus* group. *J. Bacteriol.* 181 2624–2630 10198030 10.1128/jb.181.8.2624-2630.1999 PMC93692

[B59] Rasimus S. Mikkola R. Andersson M. A. Teplova V. V. Venediktova N. Ek-Kommonen C. (2012). Psychrotolerant *Paenibacillus tundrae* isolates from barley grains produce new cereulide-like depsipeptides (paenilide and homopaenilide) that are highly toxic to mammalian cells. *Appl. Environ. Microbiol.* 78 3732–3743 22407690 10.1128/AEM.00049-12 PMC3346359

[B60] Raymond B. Wyres K. L. Sheppard S. K. Ellis R. J. Bonsall M. B. (2010). Environmental factors determining the epidemiology and population genetic structure of the *Bacillus cereus *group in the field. *PLoS Pathog* 6:e1000905. 10.1371/journal.ppat.1000905 PMC2873914 20502683

[B61] Read T. D. Turingan R. S. Cook C. Giese H. Thomann U. H. Hogan C. C. (2010). Rapid multi-locus sequence typing using microfluidic biochips. *PLoS ONE * 5:e10595. 10.1371/journal.pone.0010595 PMC2868872 20485679

[B62] Rowan N. J. Deans K. Anderson J. G. Gemmell C. G. Hunter I. S. Chaithong T. (2001). Putative virulence factor expression by clinical and food isolates of *Bacillus* spp. after growth in reconstituted infant milk formulae. *Appl. Environ. Microbiol.* 67 3873–3881 11525980 10.1128/AEM.67.9.3873-3881.2001 PMC93104

[B63] Segerman B. DeMedici D. Ehling-Schulz M. Fach P. Fenicia L. Fricker M. (2011). Bioinformatic tools for using whole genome sequencing as a rapid high resolution diagnostic typing tool when tracing bio-terror organisms in the food and feed chain. *Int. J. Food Microbiol.* 145 S167–S176 20826036 10.1016/j.ijfoodmicro.2010.06.027

[B64] Stark T. Marxen S. Rütschle A. Lücking G. Scherer S. Ehling-Schulz M. (2013). Mass spectrometric profiling of *Bacillus cereus* strains and quantitation of the emetic toxin cereulide by means of stable isotope dilution analysis and HEp-2 bioassay, *Anal. Bioanal. Chem.* 405 191–201 23079954 10.1007/s00216-012-6485-6

[B65] Stenfors Arnesen L. P. Fagerlund A. Granum P. E. (2008). From soil to gut: *Bacillus cereus* and its food poisoning toxins. *FEMS Microbiol. Rev.* 32 579–606 18422617 10.1111/j.1574-6976.2008.00112.x

[B66] Stephan R. (1996). Randomly amplified polymorphic DNA (RAPD) assay for genomic fingerprinting of *Bacillus cereus* isolates. *Int. J. Food Microbiol.* 31 311–316 8880317 10.1016/0168-1605(96)00966-x

[B67] Swaminathan B. Gerner-Smidt P. Ng L. K. Lukinmaa S. Kam K. M. Rolando S. (2006). Building PulseNet International: an interconnected system of laboratory networks to facilitate timely public health recognition and response to foodborne disease outbreaks and emerging foodborne diseases. *Foodborne Pathog. Dis.* 3 36–50 16602978 10.1089/fpd.2006.3.36

[B68] Thorsen L. Hansen B. M. Nielsen K. F. Hendriksen N. B. Phipps R. K. Budde B. B. (2006). Characterization of emetic *Bacillus weihenstephanensis*, a new cereulide-producing bacterium. *Appl. Environ. Microbiol.* 72 5118–5121 16820519 10.1128/AEM.00170-06 PMC1489381

[B69] Tourasse N. J. Helgason E. Klevan A. Sylvestre P. Moya M. Haustant M. (2011). Extended and global phylogenetic view of the *Bacillus cereus* group population by combination of MLST, AFLP, and MLEE genotyping data. *Food Microbiol.* 28 236–244 21315979 10.1016/j.fm.2010.06.014

[B70] Tourasse N. J. Helgason E. Økstad O. A. Hegna I. K Kolstø A. B. (2006). The *Bacillus cereus* group: novel aspects of population structure and genome dynamics. *J. Appl. Microbiol.* 101 579–593 16907808 10.1111/j.1365-2672.2006.03087.x

[B71] Tourasse N. J. Kolsto A. B. (2008). SuperCAT: a supertree database for combined and integrative multilocus sequence typing analysis of the *Bacillus cereus* group of bacteria (including *B. cereus,* *B. anthracis* *B. thuringiensis*). *Nucleic Acids Res.* 36 D461–D468 17982177 10.1093/nar/gkm877 PMC2238978

[B72] Tourasse N. J. Okstad O. A. Kolstø A. B. (2010). HyperCAT: an extension of the SuperCAT database for global multi-scheme and multi-datatype phylogenetic analysis of the *Bacillus cereus* group population. *Database (Oxford)* 2010 baq017. 10.1093/database/baq017 PMC2997605 20651034

[B73] Tsukamoto K. Mukamoto M. Kohda T. Ihara H. Wang X. Maegawa T. (2002). Characterization of *Clostridium butyricum* neurotoxin associated with food-borne botulism. *Microb. Pathog.* 33 177–184 12385745

[B74] Vassileva M. Torii K. Oshimoto M. Okamoto A. Agata N. Yamada K. (2006). Phylogenetic analysis of *Bacillus cereus* isolates from severe systemic infections using multilocus sequence typing scheme. *Microbiol. Immunol.* 50 743–749 16985296 10.1111/j.1348-0421.2006.tb03847.x

[B75] Wehrle E. Didier A. Moravek M. Dietrich R Märtlbauer E. (2010). Detection of *Bacillus cereus *with enteropathogenic potential by multiplex real-time PCR based on SYBR Green I. *Mol. Cell. Probes* 24 124–130 19944752 10.1016/j.mcp.2009.11.004

[B76] Wenning M. Scherer S. Naumann D. (2008). “Infrared spectroscopy in the identification of microorganisms,” in *Vibrational Spectroscopy for Medical Diagnosis,* edsDiem M. P. Griffiths R. Chalmers J. M. (Chichester: John Wiley & Sons Ltd) 71–96

[B77] Wielinga P. R. Hamidjaja R. A. Agren J. Knutsson R. Segermanm B. Fricker M. (2011). A multiplex real-time PCR for identifying and differentiating B. *anthracis* virulent types. *Int. J. Food Microbiol.* 145 S137–S144 10.1016/j.ijfoodmicro.2010.07.039 20826037

